# Aptamers and Their Significant Role in Cancer Therapy and Diagnosis

**DOI:** 10.3390/biomedicines3030248

**Published:** 2015-09-01

**Authors:** Joy Sebastian Prakash, Karunanithi Rajamanickam

**Affiliations:** Faculty of Allied Health Sciences (FAHS), Chettinad Academy of Research and Education, Kelambakkam, Chennai 603103, Tamil Nadu, India; E-Mail: sebastian5689@gmail.com

**Keywords:** aptamers, SELEX (Systematic Evolution of Ligands by EXponential enrichment), cell surface biomarkers, nanoparticles, aptasensors, cancer imaging

## Abstract

Aptamers are nucleic acid/peptide molecules that can be generated by a sophisticated, well-established technique known as Systematic Evolution of Ligands by EXponential enrichment (SELEX). Aptamers can interact with their targets through structural recognition, as in antibodies, though with higher specificity. With this added advantage, they can be made useful for clinical applications such as targeted therapy and diagnosis. In this review, we have discussed the steps involved in SELEX process and modifications executed to attain high affinity nucleic acid aptamers. Moreover, our review also highlights the therapeutic applications of aptamer functionalized nanoparticles and nucleic acids as chemo-therapeutic agents. In addition, we have described the development of “aptasensor” in clinical diagnostic application for detecting cancer cells and the use of aptamers in different routine imaging techniques, such as Positron Emission Tomography/Computed Tomography, Ultrasound, and Magnetic Resonance Imaging.

## 1. Introduction

There are numerous endemic health concerns in recent times, and one among them is cancer [1,2]. Many factors, such as viral infection, environmental factors, and abnormalities at the genetic level persuade cells to act differently at the molecular level, leading to cancer. Hence, there lies an interesting fact in identifying tools for histopathological evaluation in order to determine the presence of cancer cells among the healthy cells. Cell surface receptors differentiate cancer cells from normal cells. These cell surface receptors can be utilized as a target for detecting cancer cells and its prognosis [3,4]. Hence, exploring the presence of potential cell surface receptors will help in early cancer detection, precise pretreatment initiation, predicting the pharmacokinetics of anti-cancer drugs, and determining disease progression [5,6].

The widely adapted anti-cancer treatment regimes are chemotherapeutics [7]. Though anti-cancer treatments increase the survival rate of patients, they are delimited by poor tissue selectivity, higher systemic clearance, and reduced accumulation inside tumors [8]. Therefore, site directed delivery of chemotherapeutic drugs to tumor cells without posing toxicity to healthy tissue is a very demanding scenario and it has recently attracted researchers to explore this area [9]. Antibodies are one of the conventionally-used site-targeting molecules, however, these have been recently overthrown by novel targeting molecules, such as short peptides and aptamers [10,11]. In this review, aptamers and their selection process are described, followed by their application in clinical diagnosis and imaging. The application of aptamers, conjugated with nanoparticles and gene silencers for therapeutic purposes in prostate and colon cancer are discussed in our study. Further, we highlight the use of aptamers as imaging probes for detecting cancer cells using different imaging modalities.

## 2. Aptamers (*Aptus* + *Meros)*

Aptamers are short single stranded nucleic acids (of ~30 to ~70 nucleotides in length on average), of which the name was derived from the Latin *aptus*, meaning “to fit”, and *meros* (Greek), meaning “part”, by Andrew Ellington in 1990 [12].They fold into three-dimensional structures and bind to their targets with high specificity and affinity [13,14] and have a fixed sequence for primer binding at both the termini for amplification using Polymerase Chain Reaction (PCR) [15,16]. Craig Tuerk and Larry Gold, who were working on bactriophage T4 DNA polymerase, elaborated the process of selecting RNA ligands, which specifically binds to its target protein [17]. From then, there has been an exponential increase in the number of aptamers being used as a biotechnological tool for the validation of functions and the interaction of many proteins and ligands [18]. As a characterization tool, it can be used to interact with molecular pathway, studying the biochemical nature, which could resolve many mysteries of disease progression [19].

The discovery of aptamers was made possible due to the development of oligonucleotide screening using Systematic Evolution of Ligands by EXponential enrichment (SELEX). In this technique, repeated cycles of selection, amplification, and washing the nucleotide ligand were employed until it shows high specificity against the target [20]. Surprisingly, a conventional SELEX process starts with a random pool of 10^13^–10^15^ oligonucleotides, which are chemically synthesized as DNA libraries [21]. These chemically synthesized oligonucleotides have sequences randomly stacked at the central region with 5′ and 3′ known nucleotide bases towards both the ends. The complexity of the library increases with the randomness of the central nucleotide sequences [22]. The recent advancement in aptamer selection was Non-Equilibrium Capillary Electrophoresis of an Equilibrium Mixture (NECEEM) developed by Krylov and co-workers [23]. This approach helps to select aptamers with high affinity in lesser rounds of amplification when compared to conventional SELEX, thereby reducing time consumption [23].

As many selection processes available in consideration, the functional aspect of aptamer is conferred by a stable three-dimensional structure and that is directly related to the sequence and length of the aptamer. The specificity of aptamer increases with the complexity of three-dimensional structures, such as G-quadruplex, stems, hairpins, internal loops and bulges. The specific binding of an aptamer to its target involves hydrogen bonding, van der Waals, and electrostatic interactions [24]. Aptamers bind to their target molecule with low dissociation constant (*K_d_*) ranging from picomolar (1 × 10^−12^ M) to nanomolar (1 × 10^−9^ M) [25]. Hence, aptamers have low or no immunogenicity, providing a key advantage of pharmacokinetic modification, which leads to increased use of aptamers in therapeutic research [26]. Pegaptanib sodium (also known as Macugen), was the first RNA based aptamer approved by the US Food and Drug Administration (FDA) in December 2004, for therapeutic use. It is an anti-vascular endothelial growth factor (anti-VEGF) aptamer for humans to treat age-related macular degeneration [27].

When comparing to antibody-based immunoassays like ELISA, the use of aptamers in clinical application is gaining its momentum due to their numerous advantages, as discussed below:

1. Stability over higher temperatures: The oligonucleotides are thermally stable when compared to protein antibodies, where as aptamers do not lose their tertiary structure over many cycles of amplification at higher temperatures, and, thus, establishing a benefit for us to use aptamers in the different screening process [28,29].
2. Synthesis: Aptamers are synthesized chemically under controlled laboratory conditions, which will be useful for increased production and to avoid contamination by virus or bacteria. However, antibodies that are produced in biological conditions are mostly susceptible to viral or bacterial contamination affecting its quality [30].
3. Modification: Compared to antibodies, aptamers can be easily altered chemically, especially with signaling molecules, such as probes, nanoparticles, and fluorophores, helping in the construction of signaling based biosensors [31].
4. Low immunogenicity: Owing to their smaller size, they are least recognized by the human immune system and they easily evade it. Aptamers posses low immunogenicity and low toxicity, thus facilitating its seamless entry into biological compartments [30,32,33,34].

Apart from the above stated advantages, aptamers can be selected for various targets ranging from small molecules—organic and inorganic, drugs, products of metabolism, or even the whole cell, thereby establishing its need in a variety of fields, such as clinical diagnostics, safety of food and its products, environmental monitoring and even in defense against chemical warfare [35]. The aptamers gain their specificity, affinity and all other added advantages, by undergoing a rigorous selection procedure called SELEX, which is explained below.

## 3. SELEX

Combinatorial chemistry is an important tool for discovering or identifying new molecules that can boost research in industry, biotechnology, and pharmaceutical companies. Because of their ability to fold into secondary and tertiary structures, nucleic acids are well-suited and attractive compounds for combinatorial chemistry. Further, they have the advantage of being amplified by polymerized chain reaction (PCR) technique or transcribed *in vitro*, easily. Using SELEX as a baseline tool, nucleic acids are used to generate aptamers for different molecular targets. A chemically synthesized complex library contains oligonucleotides with random sequences of about 10^15^ molecules, and, from this library, the molecules are screened and isolated for a particular function [36].

**Figure 1:**
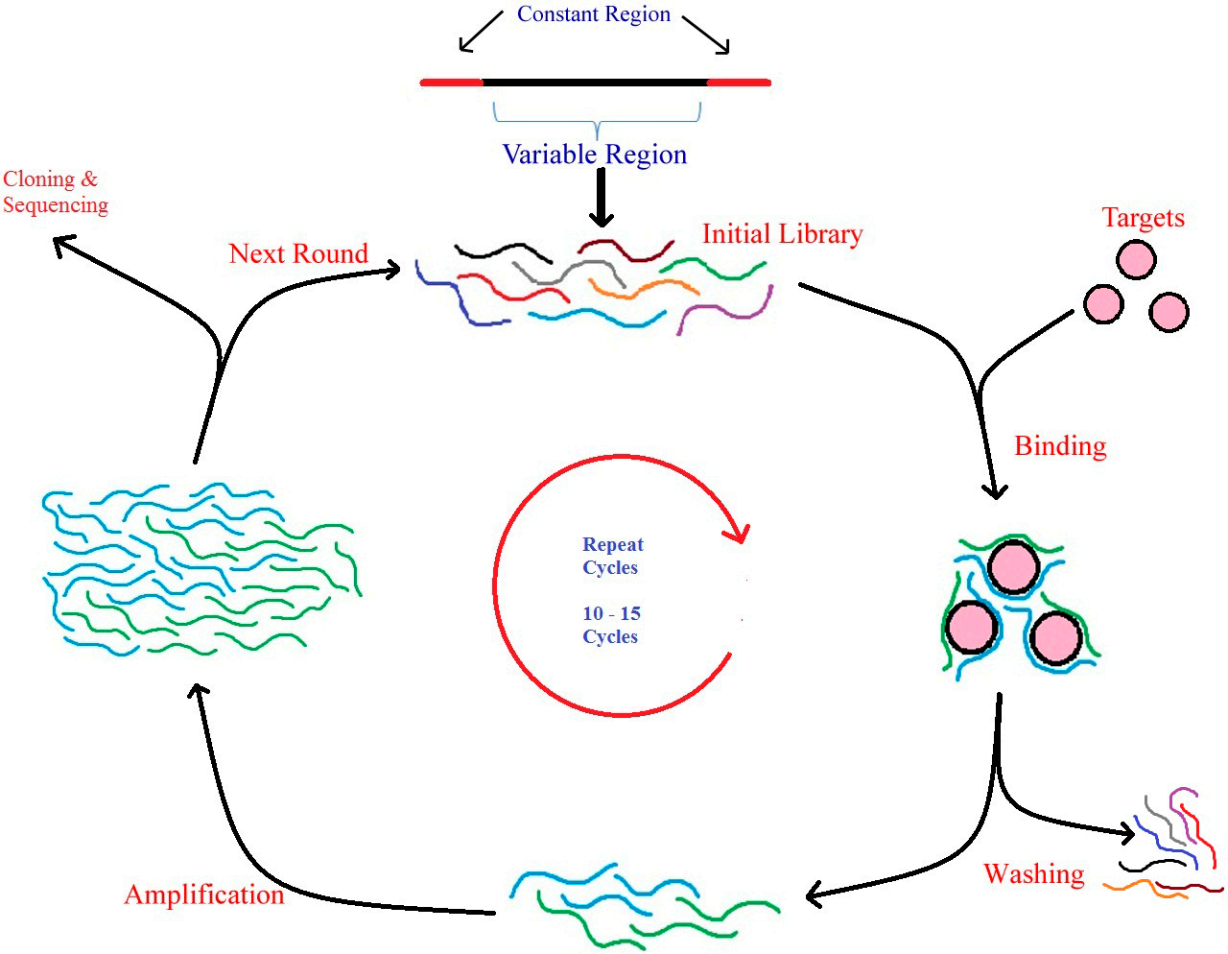
Schematic representation of SELEX process—Oligonucleotide library generation, binding, washing, amplification, cloning and sequencing of aptamers.

As shown in the Figure 1, the *in vitro* identification of aptamers, by SELEX, initially involves in the incubation of a random DNA library pool with the target molecule (that can be metal ions, organic dyes, amino acids, antibiotics, peptides, proteins, viruses, bacteria, and even whole cells). Later, the sequences that have been bound to targets are eluted and incubated with control and followed by amplification by PCR (named as DNA-SELEX) or reverse transcription (RT)-PCR (known as RNA-SELEX). This process is continuously repeated until the sequences attain specificity against its target molecules. The specificity conferred to oligonucleotides depends on the different conditions, such as concentration of the target and its properties [37], initial random DNA library [38], conditions used for selection, ratio between the target and oligonucleotides. Finally, these enriched pools of sequences with higher specificity against its targets were cloned into bacteria. The positive clones were used for sequencing to obtain the individual sequence of an aptamer [39].

### 3.1. Target Molecules for SELEX

The spatial conformations of aptamer’s folding are determined by its target molecule, and hence they are important in aptamer selection [40,41]. Since 1990, various kinds of aptamers (both RNA and DNA) were identified against different target molecules by SELEX. Many molecules such as peptides and proteins, carbohydrates, antibiotics, whole cells and even small organic and inorganic molecules have been used as targets [42]. The smallest organic molecule for which the aptamer was developed is Ethanolamine, the known smallest single stranded DNA aptamer. Ethanolamine involves in the biosynthesis of acetylcholine and has inference in diseases such as Alzheimer’s, Schizophrenia, and Ethanolaminosis [43].

The aptamer first developed against protein molecule was anti-thrombin DNA aptamer and thrombin has no biological interaction with nucleic acids. DNA aptamer against thrombin will fold into G–Quartet structures and will hinder the function of thrombin [44]. Pure soluble protein preparations were incubated with oligonucleotide libraries to use them as targets for aptamer selection. Aptamers against cell surface receptors like human tenascin-C (TN-C) [45], Human epidermal growth factor receptor-3 (HER-3), prostate-specific membrane antigen (PSMA), and many other receptors were successfully generated for cancer diagnosis and therapy [46,47].

Although small molecules and receptors were targeted, presently, aptamers are also used to combat viral infections either by inhibiting viral replication or blocking the viral entry. There is a steep increase in aptamer development for Human immunodeficiency virus HIV-1, notably, aptamers are developed for HIV glycoprotein 120 (gp120) [48]. Aptamers were developed against surface glycoprotein B and H of Human cytomegalovirus (HCMV), which facilitates the viral entry inside the host cell [49]. Aptamers, which target polymerase enzyme, specifically, a non-structural protein 5B (NS5B) has been developed for hepatitis C virus (HCV). This aptamer interfere with the transcription of viral genome, thereby preventing its replication [50,51]. The antiviral property of aptamers were explored in parallel with molecular targets that have a role in causing cardiovascular disorders (CVD). Aptamers were generated for molecular targets like von Willebrand factor (vWF) [52,53] and Factor IXa, as they are key players in causing CVD [54]. As target molecules are important, an oligonucleotide library is also a critical aspect in generating aptamers with high specificity and affinity.

### 3.2. Oligonucleotide Library for SELEX

The SELEX process starts with the chemical synthesis of DNA molecules having a complexity of 10^15^ unique sequences. This library comprises of DNA sequences with an average of 100 bases in random within the boundary of known sequences for primer hybridization at the 5′ and 3′ ends [12,14,35]. For the use of RNA as aptamers, the synthesized DNA oligonucleotide library has to be converted into RNA library before the start of the first cycle of RNA-SELEX. The success of SELEX depends on the complexity of the DNA library generated. The following are some important considerations in oligonucleotide library design, (i) the number of nucleotides in the random region; (ii) type of randomization; and (iii) the modification of DNA and RNA moieties. The structural complexity is determined by the size of random sequences, which can have long sequences for unknown targets. These longer random sequences provide a best-case scenario for identification of aptamer [36,55]. After designing and randomization of oligonucleotide library, the stability of nucleotides has to be taken care of to achieve stronger affinity and specificity.

Although DNA aptamers are stable, RNA aptamers require typical modification at 2′-OH position of the sugar moiety in order to achieve the relative stability. The ribose 2′-OH group can be replaced with a 2′-NH_2_ or 2′F group or with the substitution of 2′-*O*-methyl substituted nucleotides that confers stability to RNA aptamers and prevents its degradation from nucleases [56]. The phosphate backbone of nucleic acids also can be modified for increased stability. The replacement of non-binding oxygen group with sulfur, in the phosphodiester linkage makes the nucleic acid resistant to nuclease digestion. This kind of aptamer, with a sulfur substituted phosphate backbone, is called “thioaptamers” [57].

In addition to this, a variety of modified nucleic acids, known as “Locked Nucleic Acid (LNA)”, have been incorporated into DNA and RNA strands are presently in wide use. Aptamers having these kinds of modified nucleic acids have higher thermal stability in duplex form. LNA aptamers also exhibit higher nuclease resistance and biostability [58]. Recently, 2′-deoxy-2′-fluoro-ribonucleic acid (FNA) has been used for aptamer selection. The 2′-fluorination, modified the sugar moiety, resulting in enhanced biostability. However, they are DNA based strands, their conformation was found to be A-strand, typically seen in RNA [59]. The stable form of the oligonucleotides was fed to the next round of processing, which includes selection, amplification by PCR, and conditioning.

### 3.3. Selection, Amplification and Conditioning of Aptamers

The selection process involves in the binding of target molecules with a pool of oligonucleotides, followed by the removal of unbound oligonucleotides and the collection of bound “necessary” oligonucleotides. This helps in the generation of affinity and specificity of aptamers towards the target molecule. It is necessary that the target molecules should be exposed directly to the oligonucleotide library, where both are incubated over a period of time. For efficient selection, affinity chromatography can be used to immobilize the target molecules, which effectively separates the oligonucleotides bound to the immobilized target [60]. Alternatively, a separation method without immobilizing targets uses ultrafilteration by nitrocellulose filters with different molecular weight cutoffs [61]. These selected aptamers are subjected to amplification.

The necessity of amplification is to exponentially increase the number of target specific oligonucleotides, which are very low in concentration when compared with the initial oligonucleotide library. Apart from amplification, use of special primers helps to modify the selected oligonucleotide for the attachment of some functional groups. Functional groups bound to oligonucleotides help in detection and immobilization of those oligonucleotides. Amplification is different for DNA and RNA aptamers, where the latter has to be amplified by a reverse transcription PCR (RT-PCR) [36].

After amplification using PCR, the product has to be inducted to the next round of SELEX—“conditioning”. The product of amplification is a double stranded DNA, where it can be separated into two strands for DNA-SELEX, or it has to undergo transcription by T7 RNA polymerase for RNA-SELEX. The separation of double stranded DNA can be achieved either by a streptavidin/biotin system [62] or by performing an asymmetric PCR, which uses a higher concentration of only one primer to obtain ssDNA products [63].

### 3.4. Variations in SELEX Procedure

The advantage in the SELEX procedure lie in the flexibility of altering the working methodology, conditions for binding, and design of library. The process has also been automated to reduce time consumption. The primary objective of the modifications in the SELEX process is to obtain improved aptamers or to simplify the process [64]. The following is a brief description of the technical advancements done to the conventional SELEX process.

#### 3.4.1. Atomic Force Microscopy (AFM)—SELEX

AFM can be used to detect adhesion of affinity forces between the probe “cantilever” and the sample used [65]. Using this notable feature, SELEX was developed in tandem with AFM. In 2009, Miyachi *et al.* [66] described a method that uses Atomic Force Microscopy (AFM) along with SELEX to obtain aptamers for target molecules. This type of AFM-SELEX resulted in DNA aptamers that have higher specificity and affinity within fewer rounds of selection to their target molecule, such as thrombin.

#### 3.4.2. Automated SELEX

Automated SELEX executes all selection cycles without any intervention. Progress was monitored by measuring PCR yield, where semi-quantitative Polymerase Chain Reaction (PCR) was performed automatically, at every round of selection. This yield measurement helps to assess the bound nucleic acids to the target. Further, ultrafiltration is used for purification in automated SELEX, which effectively separates RNA from molecules of low molecular weight (such as NTPs, salts, detergents) [67].

#### 3.4.3. Cell SELEX

The whole cell can be used as targets to generate aptamers, where the molecular markers are unknown. To generate aptamers against cancer, whole cancer cells are used as targets and the aptamers are selected based on high selectivity and specificity against specific cell surface signatures. Further, the aptamers generated by whole cell SELEX can be conjugated with gold nanoparticles or quantum dots for detection of cancerous cells using colorimetric or fluorescence methods. This generation and detection method can be applied for the early diagnosis and detection of cancer [68,69].

#### 3.4.4. Capillary Electrophoresis (CE)—SELEX

Analytical chemical technique—Capillary electrophoresis (CE)—can also be incorporated in the SELEX process for achieving aptamers with a high specificity and selectivity. Mendonsa and Bowser demonstrated that CEs can be used, along with SELEX, for generating aptamers that are specific to Human immunoglobulin E (IgE). High affinity and high sensitive aptamers were generated against IgE within two rounds of selection, compared to 8–12 rounds of selection in conventional SELEX. This method proves to be versatile as it reduces time consumption without compromising the affinity of aptamers [70].

#### 3.4.5. Non-Equilibrium Capillary Electrophoresis of Equilibrium Mixtures (NECEEM)

Non-Equilibrium Capillary Electrophoresis of Equilibrium Mixtures (NECEEM), which is a kinetic capillary electrophoresis-SELEX, selects DNA aptamers in a single round of separation and amplification. Here, the targets are mixed with the DNA library and incubated to form an equilibrium mixture. When the free molecules and oligonucleotide-target complexes attains an equilibrium, they are subjected to high voltage and made to pass through gel-free capillary electrophoresis and separated by non-equilibrated separation buffer. The components of the DNA library or the targets will not be incorporated in this separation buffer, which makes it a non-equilibrium buffer. Based on the electrophoretic mobility, the target bound DNA molecules were separated from the unbound DNA and free target molecules. The target bound DNA molecules migrate to a specific electrophoresis zone between the unbound DNA and free target molecules. Finally, the target bound DNA is collected out of the capillary tube [23,71].

#### 3.4.6. FluMag—SELEX

This method has been developed based on two simple modifications—radioactive labeling was replaced by fluorescent labels, which quantify DNA, and target immobilization was achieved by magnetic beads. Target immobilization is an added advantage for easy handling, and also requires a very low concentration of targets for aptamer selection [72].

#### 3.4.7. Slow Off-Rate Modified Aptamer (SOMAmer)

A new proteomics technology, combined with aptamers, has been developed, capable of measuring proteins at very low concentration in a given biological sample. To detect proteins, DNA aptamers were modified with chemical nucleotides, which are similar to amino acid side-chains. These modified aptamers, with very slow off-rates, are selected by modified SELEX, together called Slow Off-rate Modified Aptamer (SOMAmer). Though the proteins number in the thousands in a given sample, this technology is capable of detecting at lower limits and with high reproducibility [73].

## 4. Aptamer-Based Therapy

Conventional cancer therapies include chemotherapy, radiotherapy, photodynamic, and photothermal therapy will cause detrimental side effects due to their toxicity to the healthy cells [74]. To evade this drawback of nonspecific toxicity, targeted therapy was designed, which uses antibody-based drugs. The antibody therapy also has fewer side effects, but they are limited by high production cost and immunogenicity [13,75]. Aptamer-based targeted therapy is being explored to overcome the limitations faced by antibody-targeted therapy. Thus, aptamers can be conjugated with nanoparticles and nucleic acids for efficient, targeted treatment [76].

### 4.1. Aptamer with Nanoparticles

Biodistribution and biocompatibility are also salient features of nanoparticles characterized by a large surface area, and uniform size and shape. The half-life of aptamers and the payload capacity of the drug can be improvised by conjugating with nanoparticles. The biocompatibility can be further explored with the use of liposomes with aptamers for targeted drug delivery [77]. An aptamer-targeting Prostate Specific Membrane Antigen (PSMA) was conjugated with poly lactic acid—polyethylene glycol (PLA-PEG) or with poly lactic co-glycolic acid PLGA-PEG. Here, the aptamer specifically targets biomarkers on prostate cancer cells, where PLA/PGLA helps in the encapsulation of the drug and controlled release. The attached PEG ensures the increase in half-life of the bioconjugate in systemic circulation [78]. In another study, A10, an RNA aptamer that specifically targets PSMA, was functionalized with Platinum-Pt(IV) encapsulated with PLGA-PEG and was analyzed for its pharmacokinetics and distribution. The study concluded that there was an increase in tolerated dosage level and increased circulation time in blood. This study further confirms that there was decreased toxicity to the kidneys and enhanced antitumor efficiency *in vivo* [79].

Mucin 1 (MUC1) protein is considered as one of the most important targets in cancer therapy because of its high level of expression. An anti-cancer drug, Paclitaxel (PTX), was encapsulated with PLGA and conjugated with aptamers that target the MUC1 biomarker. This drug-encapsulated aptamer enhanced *in vitro* drug delivery, specifically, to the MUC1 expressing cancer cells [80]. Further, AS1411 is a 26-nucleotide DNA aptamer, which can be used for targeting tumor cells. In research conducted by Aravind and his co-workers, this aptamer was bound with PLGA-Lecithin-PEG encapsulated with PTX. This bioconjugate exhibit sustained drug release, and internalized inside the target cells, thereby improving the *in vitro* cytotoxicity [81].

Despite PLA- and PLGA-mediated targeted therapy, metal nanoparticles are also a useful tool in targeted drug delivery systems because of properties such as stability, optical compatibility, and biocompatible nature. An RNA aptamer that specifically binds to CD30 was bound to hollow gold nanospheres (HAuNS), along with a drug called Doxorubicin (Dox), and this bioconjugate Apt-HAuNS-Dox was sensitive to pH variations. In an *in vitro* study, it selectively kills lymphoma cells, although it was incubated with several cell types in the same culture [82]. Recently, gold nanoparticles were used in photothermal therapy, which uses near-infrared laser irradiation. On the surface of gold nanorods, two specific aptamers—CSC1 and CSC13—were conjugated, which bind to both stem and non-stem cancer cells with higher affinity and specifically kills them [83]. Recently, magnetic nanoparticles have also been explored for their use in clinical imaging.

Due to biocompatibility and its degradable nature, magnetic nanoparticles are gaining importance in biological studies and one such particle is Super Paramagnetic Iron Oxide Nanoparticles (SPION). It has low *in vivo* toxicity and can be modified with targeting ligands for clinical applications [84]. A bioconjugate comprised of thermally cross-linked SPION (TCL-SPION) bound to A10 RNA aptamer and DOX were used to detect and deliver a therapeutic agent to prostate cancer cell lines. The DOX was delivered to the targeted cell with no signs of cytotoxicity [85]. In another study, SPION was conjugated with aptamer 5TR1, which specifically binds to MUC1 of C26 cell lines (colon carcinoma cells). This complex was added with Epirubicin (Epi) for chemotherapeutic effect. This Epi-Apt-SPION complex increased Magnetic Resonance Imaging (MRI) efficiency using a fat saturation technique. It is characterized by two different properties—(i) a chemical shift that showed the difference in resonance frequency between fat and water, and (ii) a difference in relaxation time T1, of adipose tissue and water [86]. This Epi-Apt-SPION complex also reduced the tumor volume after the injection of biocongucated drug [87].

Presently, RNA nanoparticles are developed for controlled folding, size growth, enhanced permeability and retention (EPR), are highly soluble without aggregation, and are thermodynamically stable in nature. Incorporation of modified nucleic acids makes the RNA nanoparticles resistant to RNase degradation [88,89,90]. The multivalent property of RNA nanoparticles helps to accommodate targeting molecules, such as aptamers and therapeutic molecules [88].

The increased application of RNA nanoparticles is evident due to numerous advantages, such as their higher thermodynamic stability, lower free energy, quaternary structure, *etc.* In physiological conditions, RNA will have a type A helical conformation (*i.e.*, with deeper major grooves and shallower minor grooves, which prevents depurination), whereas, DNA will have a type B helical conformation. This also favors RNA to be more stable in a highly acidic environment, however, DNA undergoes depurination and nuclease reduction. Another interesting feature is the self-assembly of RNA nanoparticles *in vivo* using DNA as a template. Recently, composite therapeutic moieties, such as siRNA, ribozymes, and aptamers that have been conjugated with RNA nanoparticles, are in lime-light [91].

### 4.2. Aptamer with Nucleic Acids

Gene silencing tools, such as small interfering RNA (siRNA) and microRNA (miRNA), are a new class of therapeutics, where aptamers are used as guiding molecules for delivery precisely to the cell or tissue [92]. Thus, combining aptamers with nucleic acid moieties will increase the targeting specificity of siRNA/miRNA towards the oncogenes and suppresses the overexpressing signals or genes in cancer cells [93].

#### 4.2.1. Aptamer si-RNA Therapy

The aptamer-siRNA chimera was first developed by McNamara and coworkers in order to target prostate cancer cell lines. A modified form of A10 RNA aptamer, which targets PSMA and was covalently conjugated with *Plk1* (polo-like kinase 1) siRNA or *Bcl2* (B-cell lymphoma 2) siRNA was used. This aptamer-siRNA binds specifically to cells that express PSMA on their cell surface, leading to the silencing of *Plk1* or *Bcl2* gene expression and hindering the *in vivo* tumor growth [93]. Similarly, Thiel *et al.* [94] showed an RNA aptamer that specifically target HER2, which was linked with *Bcl2* siRNA. This chimera, containing HER2-*Bcl2* siRNA, targets HER2 expressing cancer cells specifically and initiates the downregulation of the *Bcl2* gene, thus opening a new combinational strategy against cancer [94]. A recent study conducted by Wullner and coworkers [95] describes the binding two different aptamers, targeting a particular target, which can be bound to siRNA, forming a bivalent aptamer-siRNA chimera. The design consists of two RNA aptamers targeting PSMA, which were linked to eukaryotic elongation factor 2 (EEF2) siRNA. This bivalent PSMA aptamer-EEF2 siRNA was better when compared to monovalent aptamers in terms of prolonged half-life and enhanced *in vivo* tumor inhibition [95].

Bacterial virus phi29 has an important component called pRNA that helps in the DNA packaging motor and has two functional sites. These functional sites can be conjugated with siRNA, ribozymes, and aptamers, where they do not interfere with each other, and they assemble into nanostructures called RNA nanoparticles [91,96]. Recently, Zhou and co-workers [97] reported the fabrication of dual functional RNA nanoparticles. In this study, anti-HIV-1 siRNAs were specifically delivered to HIV-1 infected cells using an anti-gp120 aptamer. This pRNA-aptamer chimera binds to cells that expresses HIV gp120 and also inhibits replication of HIV-1 [97].

#### 4.2.2. Aptamer mi-RNA Therapy

Like siRNA, miRNA can also be used for targeted cancer treatment with the help of aptamers specific to the target tissue or cells. Dai and coworkers designed a chimeric complex consisting of an aptamer towards MUC1 and miRNA-29b, which targets ovarian carcinoma cells. The miRNA-29b sequence was bound to 3′ end of the MUC1 aptamer and was incubated with MUC1 expressing OVCAR-3 cells. The aptamer-miRNA complex was preferentially internalized, downregulating the DNA methyltransferase gene expression. This complex also restores PTEN expression, which induces apoptosis [98]. Similarly, in another study conducted by Esposito *et al.* [99], an oncogenic receptor called tyrosine kinase Axl was specifically targeted by an aptamer, GL21.T, and was linked with tumor suppressor let-7g miRNA. This GL21.T-let-7g complex was delivered to A549, a lung cancer cell line that expresses Axl on its surface. This bioconjugate successfully reduced the tumor cell proliferation and migration, and inhibited tumor growth in lung cancer animal models [99]. Although therapy for cancer is presently of great concern, its early diagnosis must be granted attention. 

## 5. Aptamer-Based Diagnosis

### 5.1. Sensors Using Aptamers

Many targeted therapies that use aptamer are still being researched. Sensors using aptamers are being explored for their feasible application in cancer detection. Sensors that use biological component are known as biosensors. Further, biosensors that use aptamers are called as “aptasensors”, where aptamers are the basic recognition elements [100]. Chemical modification yields aptamers that are specific for analytes or targets. Aptamers are immobilized on a platform with the presence of analytes or targets, which are exclusively used for clinical diagnosis [101,102].

#### 5.1.1. Electrochemical Aptasensors

A biological target sensing aptamer immobilized on a platform, which uses electrodes, that measures the electrochemical current variations from the binding of the target and aptamer is known as an electrochemical aptasensor. The major advantages of these aptasensors include their low cost of production, high sensitivity, possible miniaturization, and they do not require special optical instruments [103,104].

A label free, cancer cell detecting electrochemical sensor was developed using aptamer AS1411, along with a modified graphene electrode. In this technique, the aptamer will specifically detect nucleoin on the cell surfaces of cancer cells. It was conjugated with perylenetetracarboxylic acid (PTCA), which anchors the target on electrode surfaces. This electrochemical aptasensor is capable of detecting and differentiating cancer cells from normal cells [105]. The growth of a tumor and its transformation can be related to the potential cancer biomarker, called platelet-derived growth factor B chain (PDGF-BB). A low cost and sensitive electrochemiluminescence (ECL) aptasensor was developed by Chai and coworkers [106] for PDGF-BB by functionalizing gold nanoparticles (AuNP) with *N*-(aminobutyl)-*N*-ethylisoluminol (ABEI), and with aptamers as probes that detect the presence of PDGF-BB. This ECL aptasensor showed high sensitivity and specificity in the presence of its target PDGF-BB. The signal amplification by the ABEI-AuNP sensor was specific, simple, and stable, and showed the feasibility of extending this method for the clinical diagnosis of cancer [106].

#### 5.1.2. Fluorescence Aptasensors

In contrast to electrochemical aptasensors, molecules can be detected based on fluorescence, where the aptamer will be labeled with a fluorophore and the quencher. As an example, a well-known biomarker that characterizes epithelial malignancy is MUC1, which can be detected by a fluorescence aptasensor. This MUC1-targeting aptamer was fused with dye and with graphene oxide (GO) as a quencher. When the target molecule, MUC1, is absent, the quenching of the dye’s fluorescence will be higher, which shows a very low fluorescence in the background. When MUC1 is added, the fluorescence is recovered and it is detected with high sensitivity and specificity [107]. Similarly, the same GO is used as quencher for the detection of hepatocellular carcinoma, where the aptasensor selectively detects human liver cancer cell lines SMMC-7721 with higher fluorescence intensity [108].

### 5.2. Imaging Using Aptamers

Aptamers are considered the best imaging modality because of their smaller size and ability to target a broad spectrum of molecules, helping us to understand the physiological aspects of disease progression and treatment. Molecular imaging probes based on aptamers are designed by linking aptamers with fluorescent molecules or nanoparticles, where they are detected for fluorescence or bioluminescence [109]. Imaging probes with aptamers are designed based on their specificity against cellular proteins, such as nucleolin, integrins and cancer biomarkers, such as PSMA and MUC1. A novel imaging aptamer probe, known as an activatable aptamer probe (AAP), was designed by Shi and coworkers [110], where conformational changes due to aptamer-target binding generate florescence. This engineered AAP-sgc8 targeting CCRF-CEM lymphoma cells have three components (i) aptamer (which binds to CCRF-CEM cells), (ii) a linker, and (iii) a short DNA sequence having a quencher and fluorophore at both ends. The quencher will be near the fluorophore when the aptamer is in the native conformation, resulting in reduced fluorescence. When the target binds to the aptamer, the fluorophore is separated from the quencher due to conformational changes, increasing the intensity of the fluorescence [110].

#### 5.2.1. PET/CT Imaging

Based on decay characteristics and its ability to attach with ligands easily, ^64^Cu acts as an efficient radiotracer that can be applied for Positron Emission Tomography (PET) imaging [111]. AS1411 aptamer was bound to ^64^Cu and various chelators for the early detection of lung cancer based on imaging. Li *et al.* [112] used mouse H460 tumor xenograft model to assess the *in vivo* characteristics of ^64^Cu-bound aptamer using PET imaging at frequent intervals. The imaging study confirmed the presence of tumor, and the higher tumor contrast ratio was achieved within anhour. In a study conducted by Jacobson and coworkers, DNA aptamer that specifically targets tenascin-C was labeled with radioactive isotopes ^18^F and ^64^Cu for PET imaging. This tenascin-C aptamer has the advantage of low accumulation in the liver and kidneys, resulting in high tumor-to-background ratios. This labeled tenascin-C aptamer clearly distinguishes positive tenascin-C tumor from negative tenascin-C tumor. Labeling with short half-life ^18^F makes an efficient PET imaging probe, as the aptamer is short lived because of the high systemic clearance [113]. Aptamers have also been used for conventional Computed Tomography (CT) imaging, where A10 aptamer against PSMA was conjugated with gold nanoparticles, where they act as imaging contrast agents due to their higher atomic number. This aptamer-AuNP CT imaging probe showed four times higher intensity for the target cells when compared with non-target cells [114].

#### 5.2.2. Ultrasound Imaging

The most common clinical imaging modality is ultrasound imaging, but is limited by image resolution. Limitations can be eliminated by using contrast agents like nanobubbles, and they can be conjugated with aptamers for specific ultrasound imaging [115]. Microbubbles are coated with aptamers that bind to thrombin along with polymer DNA. The aptamers have sequences that help in binding with polymer DNA. When the target molecule, thrombin, binds to the aptamer, it mediates the removal of the aptamer from the polymer DNA–aptamer complex. This removal only causes the generation of ultrasound signals when they are exposed to elevated levels of the thrombin molecule. A novel microbubble, linked with an aptamer, was designed as an ultrasound contrast agent by Nakatsuka *et al.*, showing ultrasound activation only in the presence of thrombosis [116].

#### 5.2.3. Magnetic Resonance Imaging

**Figure 2:**
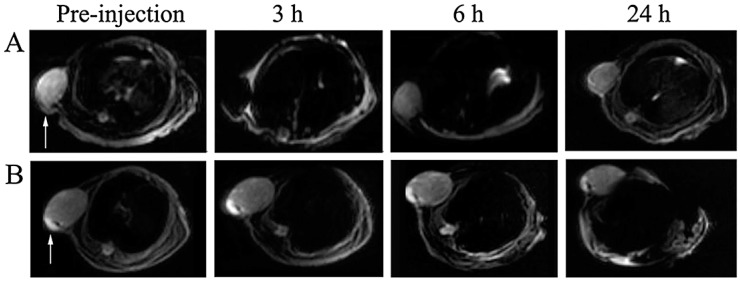
T2-weighted MRI images of xenograft-bearing nude mice injected with VEGF165-aptamer-USPIO probe (**A**) and USPIO nanoparticles only (**B**) from tail vein at a dosage of 9.08nmol/kg body weight. Arrows point to tumor xenografts. Reprinted from [117] with permission from John Wiley and Sons, copyright 2014.

Though, Magnetic Resonance Imaging (MRI) is an anatomic imaging technique, the imaging probes can be modified for use in molecular imaging. Aptamers can be conjugated with paramagnetic molecules, including gadolinium and super paramagnetic iron oxide nanoparticles (SPIONs), for MR-based imaging studies [14]. Wang and coworkers [85] described RNA aptamer A10, targeted against PSMA for prostate cancer cells and conjugated with SPION. This A10-SPION conjugate, specifically binds to PSMA expressing prostate cancer cells and were analyzed using MR imaging. In another study, vascular endothelial growth factor (VEGF) is shown to be an ideal target for cancer imaging because of its significant role in tumor metastasis. Thus, an aptamer targeting VEGF165 was chemically linked with ultrasmall super paramagnetic iron oxide (USPIO) nanoparticles. This VEGF165-aptamer-USPIO enhanced the contrast of T2-weighted imaging in the liver cancer of mice xenografts (Figure 2) [117].

**Figure 3:**
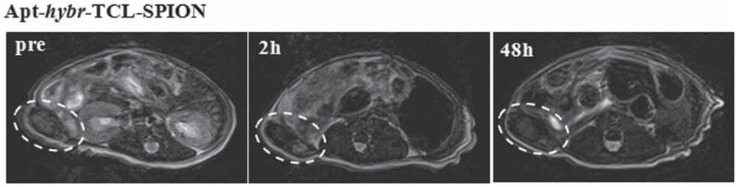
T2-weighted fast spin echo images at the level of the LNCaP tumor on the right side of the mouse taken at 0, 2, 24, 48 h after injection of Apt-hybr-TCL-SPIONs. Reprinted from [118] with permission from John Wiley and Sons, copyright 2011.

In another study by Yu and coworkers [118], thermally cross-linked SPION (TCL-SPION) was hybridized with PSMA targeting aptamers that also accommodate DOX, for both imaging and therapy. An MRI confirmed the attachment of Apt-*hybr*-TCL-SPION and it selectively delivered the drug to its target in an LNCaP xenograft mouse model (Figure 3) [118].

A recent experiment conducted by Zhang and coworkers [119], contrast agent Gd-DTPA was encapsulated inside a thermosensitive liposome (TSL) and was conjugated with the AS1411 aptamer. This TSL-AS1411 aptamer complex showed increased longitudinal relaxivity with no cytotoxic effects, even at higher Gd concentrations of 2mM. This aptamer-TSL complex, with a higher specificity and biocompatibility, can be an excellent MR imaging probe for early diagnosis [119].

## 6. Conclusions

In summary, aptamers are versatile tools for therapy and diagnosis of cancer/tumor cells. Aptamers opened a new horizon for the early detection and treatment of cancer because of its high affinity and specificity against the cell surface biomarkers. The *in vitro* selection of aptamers using conventional SELEX was successful and laborious. However, with the help of recent technical advancements, SELEX were modified to minimize time, handling costs, and human intervention. Aptamers were conjugated with conventional anti-cancer drugs and nucleic acid therapeutic molecules for anti-cancer therapy. The cross-linking of the reporter molecule with the aptamer was achieved by either physical or chemical conjugation. Thus, aptamers helps drive the therapeutic and diagnostic agents to a specific site.

## 7. Future Perspective

Though, early technology of aptamer applications has its own limitations, it has continued for more than 20 years with the integration of numerous advancements and modifications. Clinical experiments that prove that aptamers binds specifically to proteins that regulate biological functions argues in favor of aptamer based drugs and diagnostics in the near future. After Macugen, the first FDA approved drug, there were no aptamer-based drugs available in the pharmaceutical market. Many potent targets, such as HIV-1 gp120, HIV-1 RT, and many cancer cell biomarkers, are under the umbrella of research. It is not irrational to say that aptamer-based drugs for HIV and cancer will be soon available for human welfare. In future, aptamers have to be engineered for simple chemical synthesis, higher half-life circulation, enhanced tumor uptake, shortened sequences, and for efficient binding. Thus, it is sure that aptamer technology will have a strong socioeconomic impact with the possibility of early detection of cancer, *in vivo* real time cancer imaging, and personalized targeted cancer therapy.
